# Effect of dietary red grape pomace on growth performance, hematology, serum biochemistry, and meat quality parameters in Hy-line Silver Brown cockerels

**DOI:** 10.1371/journal.pone.0259630

**Published:** 2021-11-04

**Authors:** Ontiretse Jonathan, Caven Mguvane Mnisi, Cebisa Kumanda, Victor Mlambo

**Affiliations:** 1 Faculty of Natural and Agricultural Science, Department of Animal Science, North-West University, Mafikeng, South Africa; 2 Faculty of Natural and Agricultural Science, Food Security and Safety Niche area, North-West University, Mafikeng, South Africa; 3 Department of Animal Sciences, University of Pretoria, Pretoria, South Africa; 4 Faculty of Agriculture and Natural Sciences, School of Agricultural Sciences, University of Mpumalanga, Nelspruit, South Africa; University of Illinois, UNITED STATES

## Abstract

Red grape (*Vitis vinifera* L.) pomace’s (RGP) beneficial bioactive compounds could improve growth and meat quality traits in chickens and thus valorize RGP waste that is usually disposed in landfills to the detriment of the environment. This study investigated the effect of RGP inclusion in diets of Hy-line Silver Brown cockerels on physiological and meat quality responses. Five isonitrogenous and isocaloric diets were formulated by mixing a standard grower diet with RGP at 0 (G0), 15 (G15), 30 (G30), 45 (G45) and 60 g/kg (G60). A total of 250, 5-week-old cockerels (304.6 ± 6.57 g live-weight) were evenly allocated to 25 pens replicated 5 times per experimental diet. No linear and quadratic trends (*P* > 0.05) were observed for overall feed intake, body weight gain, feed conversion ratio, and meat quality traits as dietary RGP levels increased. Erythrocytes linearly decreased (*P* < 0.05), whereas mean corpuscular hemoglobin and urea linearly increased (*P* < 0.05) with RGP levels. There were significant quadratic effects for glucose, phosphorus, total protein, albumin, globulin, and cholesterol, from which a maximum RGP inclusion level was calculated to be 43 g/kg. In conclusion, dietary red grape pomace had no adverse effect on physiological parameters and meat quality traits of Hy-line Silver Brown cockerels. However, including red grape pomace beyond 43 g/kg could compromise serum biochemical parameters of the birds.

## Introduction

In rural communities, indigenous chickens play significant nutritional and socio-economic roles as major sources of animal protein and income for various households [1]. These birds are characterized by a long-life span, tolerance to harsh nutritional environment, strong adaptability, natural scavenging, and nesting habits [2,3]. These attributes have resulted in renewed efforts to preserve the genetic pool of indigenous chicken breeds [4], by developing hybrid chicken strains. Naturally, indigenous chickens are slow-growing birds with poor feed conversion ratios, low egg production and high mortality rates [4] compared to the conventional broiler and layer chickens. It is for this reason that improved chicken strains such as the Hy-line Silver Brown (HSB) are currently attracting research interest as alternative sources of dietary protein for human consumption. In comparison to indigenous birds, the improved chicken strains have better feed utilization efficiency, growth rates and high resistance against several poultry diseases [5]. To ensure sustainable intensification of improved chicken strains, there is a dire need to identify and evaluate locally available feed resources that have nutraceutical properties and have no direct food value for humans. Non-conventional feed sources such as red grape pomace (RGP) have the potential to improve growth rates, health, and feed utilization [6] while reducing feeding costs.

Red grape pomace is a by-product of grape wine-making process and is generally discarded in landfills as a waste product despite its abundance in polyphenolic compounds with potential nutritional and health benefits [7,8]. According to Spradling [9], the disposal of RGP generates serious environmental pollution, thus alternative uses of RGP need to be identified to facilitate proper management and/or utilization of this by-product. Nicodemus et al. [10] stated that RGP is a rich source of beneficial bioactive compounds with antioxidant, antimicrobial, growth-stimulating and meat-enhancing properties. Thus, the use of RGP in chicken feeds could significantly reduce the waste at wineries, while ensuring the production of healthier poultry products. Indeed, consumers are more attracted to meat that is organically produced, free of antibiotic residues and synthetic antioxidants [11]. It is important for animal producers to be able to modify the nutritional value of animal products to positively impact on human health and disease, while reducing feed costs and protecting the environment. The use of RGP in chicken diets has the potential to meet the requirements of health-conscious consumers when it comes to poultry products, because its bioactive compounds (phenolic acids, flavonoids and proanthocyanidins) are known to have health benefits [12]. Nonetheless, the presence of tannins as well as structural carbohydrates in RGP may interfere with the digestibility and utilization of nutrients in chickens [8]. In addition, too much research attention has focused on the effect of RGP in broiler chickens, with limited studies investigating its feed value in indigenous chicken diets. It is, therefore, crucial to establish the optimum dietary inclusion level of RGP that would not compromise the nutritional and health status of indigenous birds. From this viewpoint, the study was designed to determine the effect of varying inclusion levels of RGP on growth performance, haemato-biochemical indices, carcass characteristics, size of internal organs, and meat quality traits of HSB cockerels. We hypothesized that dietary RGP would improve the physiological and meat quality responses of HSB cockerels.

## Materials and methods

### Animal rights statement

The study was approved by the North-West University Animal Production Sciences Research Ethics Committee (approval number: NWU-00482-18-S5) and conformed to the guidelines for Use and Care of Research Animals.

### Research site and ingredients

The study was conducted in summer at Molelwane Research farm (25°28′0″ S, 22°28′0″ E) of the North-West University, South Africa. Sun dried red grape (*Vitis vinifera* L. var. Shiraz) pomace was procured from Blaauwklippen wine estate (Western Cape, South Africa) as described by Kumanda et al. [7]. The RGP was ground (Polymix PX-MFC 90 D, Kinematica AG, Switzerland) to pass through a 1-mm mesh screen before blending with the other feed ingredients.

### Diet formulation and analyses

A feed formulation software was used to formulate five isonitrogenous and isocaloric experimental diets (Table 1). The diets were formulated using a standard chicken grower diet mixed with 0 (G0), 15 (G15), 30 (G30), 45 (G45) and 60 g/kg (G60) RGP. The maximum inclusion level was based on results from our previous study with broiler chickens where dietary RGP at 100 g/kg compromised growth performance. The chemical composition of the RGP and the experimental diets were analyzed as described in our previous studies [7,8]. The RGP used in this study contained 966.1 g/kg dry matter (DM), 898.8 g/kg DM organic matter, 113.7 g/kg DM crude protein, 409.3 g/kg DM neutral detergent fibre, 323.3 g/kg DM acid detergent fibre, 182.3 g/kg acid detergent lignin, 70.99 g/kg DM ether extract, 1.21 AU soluble condensed tannins, and 16.43 g TAE/kg total soluble phenolics.

**Table 1:** Gross ingredient and chemical composition (g/kg *as fed*, unless stated otherwise) of experimental diets.

	^1^Diets				
^2^Ingredients	G0	G15	G30	G45	G60
Red grape pomace	0	15.0	30.0	45.0	60.0
Maize yellow	704.0	678.0	651.0	621.0	591.0
Soya oilcake (46.5%)	199.0	161.0	124.0	85.0	47.0
Full-fat soya	42.0	90.0	138.0	193.0	249.0
Gluten 60	21.00	22.00	24.00	22.00	20.00
Feed lime (50:50 mix)	14.50	14.30	14.00	13.60	13.30
Monodicalcium phosphate	7.20	7.30	7.40	7.50	7.50
Lysine (sint 78%)	2.91	2.88	2.86	2.72	2.58
Fine salt	3.12	3.16	3.20	3.29	3.37
Sodium bicarbonate	1.83	1.77	1.71	1.59	1.47
Methionine (DL 98%)	1.90	1.81	1.73	1.68	1.62
Threonine (98%)	0.34	0.32	0.30	0.28	0.25
Phytase	0.10	0.10	0.10	0.10	0.10
Choline chloride (60%)	0.80	0.80	0.80	0.80	0.80
Anticoccidial drug	0.50	0.50	0.50	0.50	0.50
Antibiotic mix	0.40	0.40	0.40	0.40	0.40
^2^Premix	0.50	0.50	0.50	0.50	0.50
**Nutritional composition**					
Dry matter	895.9	898.4	901.0	903.7	906.3
Ash	48.47	48.91	49.28	49.84	50.41
Metabolisable energy (MJ/kg)	12.14	12.14	12.14	12.14	12.14
Crude protein	179.7	179.4	179.9	179.6	179.7
Crude fat	38.71	47.11	55.44	65.07	74.80
Crude fibre	25.30	33.24	41.17	49.40	57.63
Calcium	8.18	8.21	8.21	8.20	8.19
Phosphorus	4.95	4.91	4.88	4.85	4.79
Potassium	7.12	7.25	7.39	7.61	7.84
Sodium	1.80	1.80	1.80	1.80	1.80
Chloride	3.00	3.00	3.00	3.01	3.00

^1^Diets: G0 = a standard chicken grower diet without red grape pomace; G15 = a standard chicken grower diet mixed with 15 g/kg red grape pomace; G30 = a standard chicken grower diet mixed with 30 g/kg red grape pomace; G45 = a standard chicken grower diet mixed with 45 g/kg red grape pomace; G60 = a standard chicken grower diet mixed with 60 g/kg red grape pomace.

^2^Ingredients: Phytase = Axtra phytase (100 g/t sk); Anticoccidial drug = salinomycin (12%); Antibiotic mix = olaquindox (10%); Premix: vitamin A (11000 IU), vitamin D3 (2500 IU), vitamin E (25 IU), vitamin K3 (2.0 mg), vitamin B1 (2.5 mg), vitamin B2 (4.5 mg), vitamin B6 (5.1 mg), niacin (30 mg), pantothenic acid (10 mg), folic acid (0.7 mg), biotin (0.12 g), copper sulphate (8.0 mg), potassium iodide (0.34 mg), ferrous sulphate (80 mg), magnesium sulphate (100 mg), sodium selenite (0.25 mg), and zinc sulphate (79 mg).

### Feeding trial and design

Two hundred and fifty, four-week-old Hy-Line Silver Brown male chicks were bought from Quality Breeders (PTY) LTD located in Valhalla (˗25.8001° S; 28.1554° E) in Centurion, South Africa. The birds were evenly and randomly housed in 25 pens (3.5 m L x 1.0 m W x 1.85 m H), designated as the experimental units, each carrying 10 birds. The five experimental diets were randomly allocated to the pens such that each treatment was replicated 5 times. The birds were allowed to acclimatize to the pens and adapt to experimental diets for one week before commencement of measurements from week 5 to week 16 of age. The wire-mesh pens had slatted floors that required no bedding. Feed was offered using poultry feed tubers and water was provided using normal poultry drinkers. The birds had free access to the diets and water, and rearing was conducted under natural lighting. At week 5 of age, the birds were weighed to obtain initial body weight (304.6 ± 6.57 g live-weight), and subsequently weighed weekly to determine average body weight gain (BWG). Feed was provided daily and refusals were collected before the next feeding and weighed to calculate average feed intake (FI) per bird. The BWG and FI data were used to calculate feed conversion ratio (FCR).

### Blood collection and analyses

At week 16 of age (2 days before slaughter), blood samples were collected from 10 birds randomly selected per treatment group. The blood samples were collected (2 ml) from a punctured wing vein using a 5 ml disposable syringe fitted with a 23-gauge disposable needle, where 1 ml of blood was immediately transferred to sterilized hematological tubes containing ethylene diamine tetra acetic acid, and another 1 ml transferred to serum biochemical tubes following the guidelines by Washington & van Hoosier [13]. Hematological and serum biochemical parameters were determined using an automated IDEXX LaserCyte Hematology and an automated IDEXX Vet Test Chemistry Analyzers (IDEXX Laboratories, Inc., Gauteng, South Africa), respectively.

### Carcass yield, cuts, and internal organ weights

At 16 weeks of age, all the cockerels were electrically stunned and slaughtered in a locally registered abattoir under strict hygiene. After slaughtering, carcasses from the different dietary treatments were used to determine carcass yields, hot carcass weight (HCW), and cold carcass weight (CCW). Weights of carcass cuts and internal organs were weighed using an electronic weighing scale (Explorer^®^ EX224, OHAUS Corporation, NJ, US) and expressed as a proportion of HCW (g /100 g HCW).

### Determination of meat quality parameters

According to the Commission International De I’ Eclairage [14], breast meat color coordinates: lightness (L*), redness (a*) and yellowness (b*) were measured 24 h post-mortem using a Spectrophotometer CM 2500c color-guide (Konika Minolta, Japan) set and calibrated as prescribed by the manufacturer. The color coordinates were used to calculate hue angle and chroma values as guided by Priolo et al. [15]. Simultaneously, breast meat pH was measured using a portable meat pH meter fitted spear-type electrode manufactured by Corning Glass Works (Medfield, MA, USA). The pH meter was calibrated every after 10 measurements using the standard pH solutions provided by the supplier.

For cooking losses, pieces of the breast meat samples were individually weighed and cooked until they reached an internal temperature of 75°C following the method by Honikel [16]. The cooked breast meat samples were then sheared using a Warner-Bratzler blade mounted on a Texture Analyzer (TA.XT plus, Stable Micro Systems, Surrey, UK) to determine shear force (N), a measure of meat tenderness. Breast water holding capacity (WHC) was determined using the filter-paper press method developed by Grau & Hamm [17] and calculated as follows:

$$WHC(\%)=100-[\frac{Initial\;weight-Weight\;after\;pressing}{Initial\;weight}\times 100]$$

### Statistical analysis

Overall feed intake, physiological responses, carcass characteristics, internal organs and meat quality data were analyzed using one-way ANOVA by means of the general linear model procedure of SAS version 9.4 [18], where diet was the only factor. Dose-related responses to incremental levels of RGP were evaluated using polynomial contrasts (RSREG PROC, [18]). The quadratic equation: *y = ax*^*2*^ *+ bx + c*, where y is the response variable; *c* is the intercept; *a* and *b* are the coefficients of the quadratic equation; *x* is RGP level (g/kg), was used to determine the optimal inclusion level of RGP as $\frac{-b}{2a}$. Significant trends derived from statistically similar RGP level means (based on the GLM procedure) were disregarded. For all statistical tests, significance was set at *P* < 0.05 and least squares means were compared using the probability of difference option in SAS.

## Results

### Growth performance and blood indices

Regression results showed that there were no linear or quadratic trends (*P* > 0.05) for overall BWG, FI, FCR and slaughter weights as dietary RGP levels increased (Table 2). Similarly, no dietary influences (*P* > 0.05) were observed on overall BWG, FI, FCR and slaughter weights of the cockerels.

**Table 2:** Effect of red grape pomace-containing diets on overall body weight gain, overall feed intake, overall feed conversion ratio, and slaughter weight of Hy-Line Silver Brown cockerels.

	^1^Diets						*P* value	
^2^Parameters	G0	G15	G30	G45	G60	SEM	Linear	Quadratic
Overall BWG (g)	1285.1	1285.9	1278.9	1237.4	1349.8	43.91	0.742	0.168
Overall FI (g)	5108.0	5460.0	5574.7	5378.9	5709.9	195.0	0.335	0.810
Overall FCR	3.98	4.29	4.37	4.17	4.33	0.157	0.300	0.291
Slaughter weight (g)	1599.0	1605.3	1577.7	1561.0	1569.8	52.16	0.831	0.124

^1^Diets: G0 = a standard chicken grower diet without red grape pomace; G15 = a standard chicken grower diet mixed with 15 g/kg red grape pomace; G30 = a standard chicken grower diet mixed with 30 g/kg red grape pomace; G45 = a standard chicken grower diet mixed with 45 g/kg red grape pomace; G60 = a standard chicken grower diet mixed with 60 g/kg red grape pomace.

^2^Parameters: Overall BWG = overall body weight gain; Overall FI = overall feed intake; Overall FCR = overall feed conversion ratio.

Table 3 shows that mean corpuscular hemoglobin (MCH) linearly increased [*y* = 38.5 (±6.27) + 0.772 (±0.478) *x*; R^2^ = 0.499, *P* = 0.006] in response to RGP levels. There were dietary effects (*P* > 0.05) on MCH and eosinophils. Cockerels on the control diet G0 had lower MCH (38.08 pg) compared to those on G45 and G60 diets, which were similar (*P* > 0.05). Similarly, diet G0 promoted lower eosinophils (0.655 ×10^9^/L) than diets G15 and G45, which did not differ (*P* > 0.05).

**Table 3:** Effect of red grape pomace-containing diets on hematological parameters of Hy-Line Silver Brown cockerels.

	^1^Diets						*P* value	
^2^Parameters	G0	G15	G30	G45	G60	SEM	Linear	Quadratic
Erythrocyte (×10^12^/L)	2.15	1.87	2.08	1.59	1.56	0.204	0.011	0.787
Hematocrit (%)	6.74	9.52	11.63	9.63	7.55	1.710	0.316	0.017
Hemoglobin (g/dL)	7.77	8.99	9.83	9.31	9.10	0.538	0.003	0.009
MCV (fL)	31.6	53.6	55.37	62.05	49.98	9.530	0.111	0.104
MCH (pg)	38.1^a^	52.5^a^^b^	52.5^a^^b^	60.1^b^	62.6^b^	5.490	0.006	0.538
MCHC (g/dL)	134.4	90.3	94.5	110.2	122.7	18.23	0.707	0.023
Reticulocytes (K/μL)	75.3	74.1	81.3	78.1	18.8	31.91	0.335	0.983
Leukocytes (×10^9^/L)	207.6	213.2	204.9	229.4	224.7	32.69	0.900	0.842
Neutrophils (×10^9^/L)	5.60	28.80	11.80	20.20	17.0	5.93	0.471	0.550
Lymphocytes (×10^9^/L)	185.7	161.5	142.7	188.7	156.0	43.24	0.999	0.823
Eosinophils (×10^9^/L)	0.66^a^	3.01^b^	1.99^a^^b^	3.07^b^	1.60^a^^b^	0.476	0.183	0.151
Platelet volume (%)	6.76	12.45	5.38	4.18	7.86	2.810	0.620	0.753
PDW (%)	21.20	26.45	21.27	18.73	18.53	2.570	0.120	0.808

^a,b^ In a row, means with common superscripts do not differ (*P* < 0.05).

^1^Diets: G0 = a standard chicken grower diet without red grape pomace; G15 = a standard chicken grower diet mixed with 15 g/kg red grape pomace; G30 = a standard chicken grower diet mixed with 30 g/kg red grape pomace; G45 = a standard chicken grower diet mixed with 45 g/kg red grape pomace; G60 = a standard chicken grower diet mixed with 60 g/kg red grape pomace.

^2^Parameters: MCV = mean corpuscular volume; MCH = mean corpuscular hemoglobin; MCHC = mean corpuscular hemoglobin concentration; PDW = platelet distribution width.

There were quadratic trends (*P* > 0.05) for glucose, symmetric dimethylarginine (SDMA), phosphorus, total protein, albumin, globulin, and cholesterol in response to dietary RGP levels (Table 4). Serum urea linearly increased [*y* = 0.76 (±0.025) + 0.001 (±0.002) *x*; R^2^ = 0.272, *P =* 0.028] as RGP levels increased.

**Table 4:** Responses of serum biochemical parameters in Hy-Line Silver Brown cockerels fed diets containing incremental levels of red grape pomace.

Parameters	Regression equations	R^2^	*P* value	Optimum
Glucose	*y* = 20.7 (±1.15)– 0.32 (±0.110) *x* + 0.004 (±0.0006) *x*^2^	0.366	0.008	40.0
^1^SDMA	*y* = 103.1 (±8.21)– 2.64 (±0.051) *x* + 0.036 (±0.009) *x*^2^	0.571	0.001	36.7
Phosphorus	*y* = 3.30 (±0.250)– 0.043 (±0.018) *x* + 0.001 (±0.0002) *x*^2^	0.273	0.038	43.0
Total protein	*y* = 82.6 (±0.689)– 0.945 (±0.488) *x* + 0.017 (±0.0073) *x*^2^	0.353	0.027	27.8
Albumin	*y* = 24.7 (±1.15)– 0.296 (±0.075) *x* + 0.004 (±0.0016) *x*^2^	0.325	0.014	37.0
Globulin	*y* = 55.4 (±5.40)– 0.690 (±0.382) *x* + 0.015 (±0.006) *x*^2^	0.472	0.021	23.0
Cholesterol	*y* = 5.18 (±0.313)– 0.057 (±0.022) *x +* 0.001 (±0.0003) *x*^2^	0.341	0.013	28.5

^1^SDMA = symmetric dimethylarginine.

Table 5 shows that there were no dietary effects on creatinine, calcium, alkaline phosphatase (ALKP), gamma glutamyl transferase (GGT), and total bilirubin. Cockerels on diet G60 had higher glucose (19.98 mmol/L) than those on diets G30 and G45, which did not differ (*P* > 0.05). Diet G0 promoted higher SDMA (85.63 μg/dL) than diets G30 and G45, which were similar (*P* > 0.05). Nonetheless, birds on diet G0 had the same (*P* > 0.05) SDMA as those in diets G15 and G60. Serum urea was lowest (0.69 mmol/L) in the control group than in diets G15, G45 and G60, which did not differ (*P* > 0.05). Diet G30 promoted the least phosphorus (2.23 mmol/L) than diet G15 (3.08 mmol/L) but had similar (*P* > 0.05) phosphorus levels as the cockerels on the other treatments. Similarly, diet G30 promoted the lower total protein (68.20 g/L) than diet G60 (91.30 g/L) but had the same (*P* > 0.05) total protein as the cockerels on the other treatments.

**Table 5:** Effect of red grape pomace-containing diets on hematological parameters of 16-week-old Hy-Line Silver Brown cockerels.

	^1^Diets						*P* value	
^2^Parameters	G0	G15	G30	G45	G60	SEM	Linear	Quadratic
Glucose (mmol/L)	16.64^a^^b^	19.23^a^^b^	15.02^a^	15.48^a^	19.98^b^	1.105	0.915	0.008
SDMA (μg/dL)	85.63^c^	80.20^b^^c^	48.50^a^	53.20^a^^b^	77.30^b^^c^	6.960	0.071	0.001
Creatinine (μmol/L)	12.13	12.50	9.60	14.90	18.50	2.286	0.156	0.083
Urea (mmol/L)	0.69^a^	0.80^b^	0.78^a^^b^	0.82^b^	0.83^b^	0.023	0.028	0.766
Phosphorus (mmol/L)	2.63^a^^b^	3.08^b^	2.23^a^	2.47^a^^b^	2.93^a^^b^	0.192	0.352	0.038
Calcium (mmol/L)	3.34	3.21	2.98	3.01	3.64	0.210	0.970	0.004
Total protein (g/L)	73.25^a^^b^	73.20^a^^b^	68.20^a^	72.40^a^^b^	91.30^b^	5.400	0.117	0.027
Albumin (g/L)	21.00^a^^b^	22.60^a^^b^	19.30^a^	19.00^a^	23.40^b^	1.145	0.720	0.014
Globulin (g/L)	52.38^a^^b^	46.40^a^	48.90^a^	52.80^a^^b^	67.90^b^	4.124	0.014	0.021
ALT (U/L)	25.88^a^^b^	34.70^b^	25.30^a^^b^	15.70^a^	20.0^a^^b^	4.391	0.059	0.965
ALKP (U/L)	266.8	342.9	399.7	431.5	373.4	102.8	0.489	0.657
GGT (U/L)	49.00	35.60	43.00	43.70	49.50	5.041	0.312	0.311
Total bilirubin (μmol/L)	8.50	18.80	10.20	7.20	12.30	5.171	0.502	0.722
Cholesterol (mmol/L)	4.77^a^^b^	4.63^a^^b^	4.32^a^	4.26^a^	5.24^b^	0.224	0.531	0.013

^a,b,c^ In a row, means with common superscripts do not differ (*P* < 0.05).

^1^Diets: G0 = a standard chicken grower diet without red grape pomace; G15 = a standard chicken grower diet mixed with 15 g/kg red grape pomace; G30 = a standard chicken grower diet mixed with 30 g/kg red grape pomace; G45 = a standard chicken grower diet mixed with 45 g/kg red grape pomace; G60 = a standard chicken grower diet mixed with 60 g/kg red grape pomace.

^2^Parameters: ALT = alanine transaminase; ALKP = alkaline phosphatase; GGT = gamma glutamyl transferase; SDMA = symmetric dimethylarginine.

Cockerels on diet G60 had higher globulin (67.9 g/L) than diets G15 and G30, which were similar (*P* > 0.05). Diet G60 promoted higher albumin (23.4 g/L) than diets G30 and G45, which did not differ (*P* > 0.05). Diet G15 had higher alanine transaminase (ALT) (34.70 U/L) than G45 diets (15.7 U/L). Cockerels on diet G60 had higher cholesterol (5.24 mmol/L) than diets G30 and G45, which were similar (*P* > 0.05). Nonetheless, the control diet G0 promoted similar (*P* > 0.05) glucose, phosphorus, total protein, albumin, globulin, ALT, and cholesterol levels as the RGP-containing diets.

### Carcass, visceral organs, and meat quality

Neither quadratic nor linear effects (*P* > 0.05) were observed for all carcass characteristics and internal organs as RGP levels increased (Table 6). Likewise, there were no significant dietary influences on carcass traits and visceral organs, except on proventriculus and jejunum. Cockerels on the control diet G0 had similar (*P* > 0.05) proventriculus and jejunum as those in diets G15, G30 and G60. However, diet G15 promoted the lighter (*P* < 0.05) proventriculus and jejunum weights compared to diet G45.

**Table 6:** Effect of red grape pomace-containing diets on carcass characteristics and visceral organ weights (g /100 g HCW) of Hy-Line Silver Brown cockerels.

	^1^Diets						*P* value	
Parameters	G0	G15	G30	G45	G60	SEM	Linear	Quadratic
Carcass yield (%)	70.10	70.52	72.44	71.23	70.24	1.233	0.194	0.625
Warm carcass (g)	1120.4	1131.8	1141.5	1112.1	1100.8	36.99	0.940	0.658
Cold carcass (g)	1078.7	1089.3	1105.2	1072.6	1074.6	35.19	0.664	0.359
Breast	12.96	11.76	11.40	13.96	11.60	1.355	0.150	0.115
Drumstick	7.49	7.63	7.80	7.80	7.72	0.102	0.633	0.208
Thigh	6.96	7.01	7.08	7.35	7.34	0.149	0.007	0.828
Wing	6.40	6.44	6.58	6.43	6.63	0.125	0.452	0.278
Gizzard	3.06	3.17	3.24	3.04	3.10	0.058	0.544	0.081
Liver	2.67	2.53	2.51	2.48	2.40	0.080	0.087	0.183
Spleen	0.35	0.40	0.36	0.42	0.33	0.034	0.034	0.101
Proventriculus	0.61^a^^b^	0.59^a^	0.65^a^^b^	0.68^b^	0.67^a^^b^	0.024	0.136	0.459
Duodenum	1.43	1.52	1.50	1.74	1.46	0.089	0.739	0.172
Jejunum	2.07^a^^b^	1.90^a^	2.06^a^^b^	2.35^b^	2.19^a^^b^	0.076	0.150	0.931
Ileum	1.40	1.23	1.30	1.46	2.69	0.460	0.800	0.150
Caecum	1.23	1.16	1.20	1.39	1.30	0.063	0.544	0.868

^a,b^ In a row, means with common superscripts do not differ (*P* > 0.05).

^1^Diets: G0 = a standard chicken grower diet without red grape pomace; G15 = a standard chicken grower diet mixed with 15 g/kg red grape pomace; G30 = a standard chicken grower diet mixed with 30 g/kg red grape pomace; G45 = a standard chicken grower diet mixed with 45 g/kg red grape pomace; G60 = a standard chicken grower diet mixed with 60 g/kg red grape pomace.

Table 7 shows that there were no significant linear or quadratic trends for meat quality parameters in response to increasing dietary RGP levels. There were significant dietary effects only on WHC, where meat from cockerels in diet G0 had a lower WHC (93.39%) than meat from those in diet G45 (97.45%). However, the control diet G0 promoted similar (*P* > 0.05) WHC as diets G15, G30 and G60.

**Table 7:** Effect of red grape pomace-containing diets on meat quality traits of Hy-line Silver Brown cockerels.

	^1^Diets						*P* value	
Parameters	G0	G15	G30	G45	G60	SEM	Linear	Quadratic
pH	6.46	6.58	6.54	6.58	6.46	0.101	0.748	0.397
Lightness (L*)	54.66	53.25	55.16	54.80	52.77	1.597	0.955	0.138
Redness (a*)	4.33	4.51	3.87	3.02	4.25	0.683	0.243	0.735
Yellowness (b*)	5.99	8.44	7.21	6.56	7.25	0.741	0.413	0.871
Chroma	7.49	9.59	8.20	7.24	8.56	0.875	0.947	0.908
Hue angle	0.97	1.07	1.08	1.13	1.06	0.058	0.043	0.668
Shear force (N)	8.23	6.61	7.78	8.18	8.05	1.206	0.828	0.650
Cooking loss (%)	30.76	30.03	33.95	31.92	30.90	1.503	0.316	0.325
^2^WHC (%)	93.39^a^	96.14^a^^b^	95.24^a^^b^	97.45^b^	96.29^a^^b^	0.845	0.134	0.351

^a,b^ In a row, means with common superscripts do not differ (*P* > 0.05).

^1^Diets: G0 = a standard chicken grower diet without red grape pomace; G15 = a standard chicken grower diet mixed with 15 g/kg red grape pomace; G30 = a standard chicken grower diet mixed with 30 g/kg red grape pomace; G45 = a standard chicken grower diet mixed with 45 g/kg red grape pomace; G60 = a standard chicken grower diet mixed with 60 g/kg red grape pomace.

^2^WHC: Water holding capacity.

## Discussion

Red grape pomace can potentially be used as a functional feed ingredient in animal nutrition because of its high levels of bioactive compounds (phenolic acids, simple flavonoids, stilbenes, and proanthocyanidins) with beneficial antioxidant and antimicrobial properties [19]. However, the presence of structural carbohydrates and phenolic compounds in this by-product may limit its utilization in chicken diets [8,20]. Considering this, it is imperative to investigate the maximum tolerance level of RGP inclusion in diets of Hy-Line Silver Brown cockerels to optimize nutrient utilization, growth performance, health, and meat quality. In this study, neither linear nor quadratic trends were observed for overall feed intake of the cockerels in response to varying levels of RGP. These findings are similar to those of Francesch et al. [21] who demonstrated that inclusion of 50 g/kg of grape seed in the diet of Penedes chicken did not significantly affect feed intake. These results further corroborate the results by Alm El-Dein et al. [22] where dietary supplementation with up to 40 g/kg grape pomace did not affect feed intake in Inshas strain chickens. This suggests that the inclusion of RGP as a functional ingredient in chicken diets does not compromise the palatability of the diets. There were no significant dietary influences on overall BWG and FCR, which is consistent with the findings of Francesch et al. [21] and Alm El-Dein et al. [22] who reported no differences on growth performance of chickens fed grape pomace diets. Similarly, Lichovnikova et al. [23] reported that the diet containing RGP did not have any significant effect on growth or FCR of broiler chickens. In addition, the inclusion of RGP at 20 g/kg had no effect on FCR in molted layer hens [24].

Whole blood count is commonly used to monitor the pathophysiological and general health statuses of the birds in response to nutritional and environmental factors, infectious diseases, and stress. Erythrocyte, hemoglobin and MCH are indicators of the amount of oxygen received by body tissues [13]. Results from the current study show that there was a significant linear increase on MCH in response to dietary inclusion of RGP. This signifies the potential of RGP to increase the amount of hemoglobin per red blood cell and thus enhance oxygen transportation to tissues of the chicken. Indeed, chickens fed the RGP-containing diets had higher MCH when compared to those in the control group. It is, however, important to note that MCH values remained within the normal range for chickens [25]. Furthermore, cockerels on the control treatment had the lowest eosinophil levels while those fed the RGP-containing diets had the highest, which could have been an allergic response to some of the secondary metabolites present in RGP [26]. It is also worth noting that eosinophils are reported to exhibit diurnal changes. Leukocytes and neutrophils, which are reported to increase as a response mechanism towards infection and stress, were not significantly influenced by the inclusion of dietary RGP in the diets. Due to the presence of antinutritional compounds in RGP, it was expected that the whole blood count of the cockerels fed with the RGP-containing diets would be negatively affected. Nonetheless, the blood indices were observed to be within the normal range for healthy chickens implying that RGP had no negative post-ingestive feedback.

Serum biochemistry is a reliable clinical tool widely used to monitor any changes in response to internal and exogenous factors [27]. In this study, quadratic trends were observed for glucose, SDMA, phosphorus, total protein, albumin, globulin, and cholesterol in response to increasing levels of RGP levels. This confirms that higher inclusion levels of RGP beyond 43 g/kg can compromise the health status of the birds. Cockerels on the highest RGP inclusion level had high glucose levels, which was surprising because the high levels of cellulose and hemicellulose in the RGP was expected to reduce energy density and utilization, and thus causing a decline in glucose levels. Furthermore, RGP has a protective role in the β-cell functions of the pancreas, which is thought to reduce glucose levels due to the antioxidative effect of proanthocyanidins in GP [7]. Moreover, research indicates that some phenolic compounds in grape seed may inhibit sodium-dependent glucose transport, increasing insulin resistance and reducing glucose absorption in the digestive tract [28]. Thus, the high levels of glucose could be a result of increased liver activity to detoxify amounts of secondary plant metabolites in RGP. These results were consistent with those of Khodayari and Shahriar [29], who reported that the inclusion of grape pomace at 60 g/kg in broiler diets elevated glucose levels due to high nitrogen-free extract (glucose and fructose) in the grape pulp. Urea levels were also high in cockerels fed with 60 g/kg of RGP diets when compared to those in the control group, which could have been influenced by the high total protein concentration. This is because there is a positive correlation between total protein and its constituents (urea, creatinine, bilirubin, and globulin). Similarly, no variation in total protein concentrations in the serum of chickens fed RGP-containing diets was reported [30]. High levels of liver enzymes (e.g., GGT, ALT and ALKP) above normal ranges signify hepatocellular degeneration [13]. In this study, the inclusion of dietary RGP did not alter the liver enzymes further demonstrating that phenolics and other antinutrients in RGP did not negatively affect the health status of the birds. Cockerels on diet 60 g/kg RGP had a high cholesterol level, in contrast to Khodayari and Shahriar [29] who reported a decline in cholesterol levels in broiler chickens fed diets with RGP. The fibre in RGP was expected to result in a reduction of the cholesterol level through the absorption of bile acids and various lipids [31].

The ultimate measure of profitability in any poultry enterprise is the volume of meat of high quality. Nonetheless, the inclusion of dietary RGP had no effect on all carcass characteristics and some organs. These findings agreed with those of Aditya et al. [32], who reported that supplementation of RGP in diets of laying hens did not show any effect on carcass traits and internal organ sizes. However, the inclusion of dietary RGP linearly increased the size of the spleens, which could explain the linear increase observed for MCH. Spleen enlargement indicates an anatomical adaptation to fight against infectious or inflammatory diseases and stabilizes both red and white blood cells. These results are similar to those of Brenes et al. [19] who found that the addition of RGP up to 60 g/kg did not affect liver and pancreas weight but affected the spleen weights. Cockerels fed the control treatment had similar proventriculus, gizzard, and jejunum sizes as those in the RGP-containing diets. Theoretically, the size of the intestines was expected to be longer in the RGP cockerels as an adaptive mechanism to utilize fibre and enhance digestion.

In previous studies, the use of RGP in chicken feeds was reported to improve product quality [33,34]. Contrastingly, the inclusion of varying levels of RGP had no significant linear or quadratic effects on all meat quality parameters. This was surprising because the presence of anthocyanins in RGP [35] were expected to improve the color and appearance of the meat. Only water holding capacity was influenced by the inclusion of dietary RGP with meat from cockerels on the control diet retaining less water than meat from those in diet G45. Nonetheless, the control diet promoted similar WHC as diets G15, G30 and G60. It is, however, not clear what might have caused this variation on WHC, thus more research is required to understand the effect dietary RGP in chicken meat. Overall, dietary RGP did not compromise overall feed intake, growth performance, hematological parameters, carcass traits, internal organs, and meat quality responses of the chickens. However, levels beyond 43 g/kg of dietary RGP could negatively affect serum biochemical indicators of the birds.

## Supporting information

S1 File (XLSX)
